# Melatonin's oncostatic effects in experimental breast cancer: A systematic review of dose-response and apoptotic mechanisms in animal models

**DOI:** 10.1016/j.clinsp.2026.100888

**Published:** 2026-02-25

**Authors:** Mayara Souza Alves, Luciana Lamarão Damous, Isaque da Silva Ferreira, Cecília da Silva Ferreira, Isabel Cristina Esposito Sorpreso, José Cipolla-Neto, Edmund Chada Baracat, José Maria Soares

**Affiliations:** a Laboratório de Ginecologia Estrutural e Molecular (LIM-58), Disciplina de Ginecologia, Departamento de Obstetrícia e Ginecologia, Faculdade de Medicina, Universidade de São Paulo (USP), São Paulo, SP, Brazil; b Laboratório de Neurobiologia, Departamento de Fisiologia e Biofísica, Instituto de Ciências Biomédicas, Universidade de São Paulo (USP), São Paulo, SP, Brazil

**Keywords:** Melatonin, Breast cancer, Cell migration, Apoptosis, Systematic review

## Abstract

Breast cancer remains a major health concern for women because of its prevalence and high morbidity and mortality rates. Metastasis represents the most severe prognosis, and many studies have been conducted to understand the mechanisms involved in cell migration and the factors influencing this process.

**Objectives:**

To evaluate the effects of melatonin on breast cancer cells through a systematic literature review.

**Method:**

Systematic review of the literature was carried out through PubMed, Embase and Web of Science as scientific search platforms using Population, Intervention, Comparison, and Outcome (PICO) with the keywords (P) “rat” or “murine”; (I) “melatonin’’, “pineal’’ and “indolamine’’; (C) “breast cancer model’’, “mammary cancer model’’ and “breast tissue’’ and (O) “proliferation” and “cell migration.” The inclusion criteria were full-text, open-access articles in Portuguese, English, or Spanish that focused on the antioxidant and proapoptotic actions of melatonin on breast cancer cells. The exclusion criteria were articles published >20-years ago, those with missing methodological information, and those not describing the dosage and specificity of the research product used. The findings of each included study were structured in tables with details about the sample size, dose of melatonin, and route of administration. The quality of evidence was assessed using the Cochrane risk-of-bias tool. This study was registered in PROSPERO (n° CRD42025635531).

**Results:**

A total of 257 articles were selected from the databases; 25 were read in full, resulting in 10 articles selected for this systematic review, with a total population of 756 animals studied. From the studies analyzed, six showed a reduction in tumors in correlation with the apoptosis process. This review shows that melatonin can inhibit tumorigenesis in three studies with the lowest risk of bias, which reinforces the oncostatic action of melatonin. However, several studies have combined other substances to evaluate their oncostatic action, making it difficult to infer the mechanisms involved. Further studies are necessary to elucidate the mechanisms through which melatonin acts in mammary carcinogenesis.

**Conclusion:**

The literature shows that in animal models of breast tumors, high doses of melatonin can induce tumor reduction by cell death through apoptosis. However, further studies are required to understand the mechanisms by which this hormone acts in mammary carcinogenesis.

## Highlights

• The high mortality rate associated with breast cancer makes it an important neoplasm.
• Melatonin may modulate breast cancer progression, influencing proliferation and apoptosis.
• Melatonin inhibited tumorigenesis in three studies with the lowest risk of bias.

## Background

The high mortality rate associated with breast cancer makes it an important neoplasm in medicine. Thus, investigations have aimed to reduce mortality through various therapeutic strategies, including endocrinological strategies. In addition to estrogen, melatonin can influence tumor development and progression.^1^, ^2^, ^3^, ^4^

In breast cancer cells, migration and invasion are associated with rapidly forming specialized plasma membranes and extracellular matrix structures. Furthermore, the rearrangement of the cytoskeleton facilitates cell migration. In this context, the oncostatic action of melatonin as a reduction in proliferation and the probability of metastasis has been reported by several investigators in breast cancer.^5^^,^^6^ However, this has not yet been fully confirmed.

Data from the literature show that melatonin could have an antagonistic effect on estrogen action on MCF-7 cells’ growth due to its action on Estrogen Receptors Alpha (ERα). The relationship between melatonin and estrogenic effects has also been reported in granulosa cells,^7^^,^^8^ corroborating its importance in different cell populations, especially in proliferative functions. Furthermore, melatonin also acts in programmed cell death (apoptosis); however, depending on the cell type, it can negatively or positively influence this pathway. Another way melatonin can influence tumor growth is through angiogenesis, a crucial process for the nutritional and oxygen supply to the cells, and therefore for the successful colonization of other sites during metastasis.^9^^,^^10^

In this context, it is necessary to consolidate information related to the role of melatonin in breast cancer cell proliferation and motility. Thus, the objective of this review is to summarize the literature on the effects of melatonin on breast cancer in the last 20-years, *in vivo* studies, to contribute to future studies to better elucidate the role of this indoleamine, not only in this area of oncology but also in other tumor types.

## Methods

The present systematic review was performed according to the Preferred Reporting Items for Systematic Reviews and Meta-analyses (PRISMA) guidelines^11^ and registered in PROSPERO in February 2025 (ID n° CRD42025635531).

### Literature search strategy

The PubMed, Embase, and Web of Science databases were used to select articles. The keywords used for the search were based on the Population, Intervention, Comparison, and Results (PICO) criteria, considering relevance, specificity, and frequency, allowing the expansion of searches for subsequent selection of articles of interest. For Population (P), the keywords “rat” or “murine” were used. For Intervention (I), the search included the terms “melatonin,” pineal,’’ and” indolamine” or “indoleamine.” For Comparison (C), the words “breast cancer model,’’ “mammary cancer model,” and “breast tissue’’ were used. And for Outcome (O), the term “cell migration” was adopted. Boolean operators “AND” and “OR” combined the search English terms. Although not included in the PICO, the authors consider it important to include the type of study (systematic review) in the keywords. The articles searched for in the databases included studies on melatonin alone or in combination with other drugs in animal models of mammary cancer.

### Eligibility criteria

#### Inclusion

The inclusion criteria were open-access articles in Portuguese, English, French, Italian, German, and Spanish published between 1994 and July 2024 regarding experimental studies showing the effects of melatonin, alone or conjugated, on breast cancer cells *in vivo*.

#### Exclusion

Studies with methodological inadequacy, without dosage values or specificity of the research product, and editorials or review articles were excluded when reviewing the titles and abstracts. There were no restrictions on the sample size.

### Selection

The selection phase was divided into two distinct stages: the initial screening by reading the title and abstract, and the final selection to identify experimental studies in rats that used melatonin and to evaluate the effects of its actions on breast cancer compared to a vehicle group. Once the abstracts of the articles were evaluated and the eligibility criteria were applied, screening was performed by two reviewers who worked independently (LHCMB and ISF). Duplicate articles were screened and deleted. In cases of persistent disagreement, a third reviewer (JMSJ) was consulted for the final decision. Studies that met the inclusion criteria were selected for full reading, and further analysis of the results was performed.

After selecting the articles and fully reading the data, the included studies were collected to extract relevant information from each publication and prepare the data for analysis. A table was created with details regarding the sample size, melatonin dose, and route of administration. This analysis was performed according to the PRISMA statement.^11^

The data obtained from the selected studies were tabulated, including authors, year, and place of publication, treatment time, routes of administration, and dosage of melatonin, isolated or conjugated melatonin, time of administration, presence of tumor reduction, molecular biology techniques used, and the number of animals. To identify the study heterogeneity, the authors assessed additional data, including variations in dosing, administration routes, and experimental models. Given the methodological diversity (e.g., melatonin combined with other substances, differing tumor induction methods), a meta-analysis was deemed inappropriate.

The authors assessed the risk of bias in the included studies using Cochrane's revised risk of bias tool and the Systematic Review Center for Laboratory Animal Experiments (SYRCLE) tool for animal trials.^12^ The domain questions included selection, performance, detection, attrition, and reporting. The domain questions are as follows:

D1) Was the sequence generation method previously defined and applied to allocate animals?
D2) Were the groups initially paired (with similar baseline characteristics), or was a subsequent adjustment made for analysis purposes?
D3) Was there an adequate blinding criterion to avoid predictions before and after allocating animals into groups?
D4) Were the animals randomly allocated during the experiment?
D5) Animal handlers and researchers were blinded to the intervention received by the animals during the experiment.
D6) Were the animals selected randomly to evaluate the results?
D7) Was the outcome assessor blinded?
D8) Are incomplete results (losses and exclusions during the experiment) properly addressed?
D9) Is the study free from biased reporting of results?
D10) Is the study free of other problems that may result in a high risk of bias?

The risk of bias assessment was carried out independently by two reviewers, and in case of discrepancies, a third reviewer was consulted, judging 'risk of bias' (low risk, some concerns, and high risk) for each specific result. The score in each domain was assigned according to the assessed risk: low risk = 2, moderate risk = 1, and high risk or lack of information = 0.

## Results

**Fig. 1:**
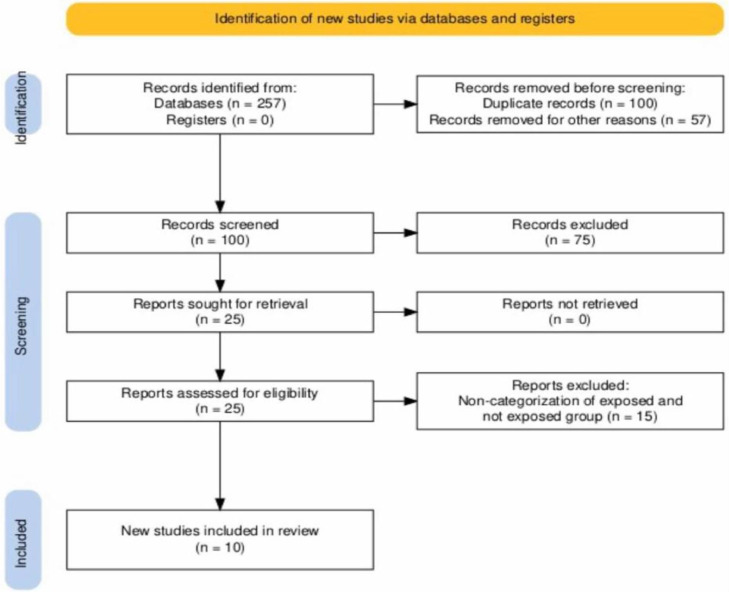
Flowchart of articles identification via databases and records ‒ Systematic review selection criteria.^11^.

The article selection algorithm is illustrated in Fig. 1. A total of 257 articles were evaluated in the databases, and 100 were excluded from the publication date filter. The titles and abstracts were read, and 132 studies were excluded based on the exclusion criteria of this systematic review. Of these, 25 manuscripts were included, but 15 were excluded from the final analysis, resulting in 10 being selected.^13^, ^14^, ^15^, ^16^, ^17^, ^18^, ^19^, ^20^, ^21^, ^22^

When using the SYRCLE tool, a total score above 12 was indicated as a low risk of bias; between 12 and 8, the data were classified as moderate risk; and below eight, the data were considered high risk of bias. Of the 10 studies included in this review, five were considered to have a low risk of bias, while another four and two studies were classified as having medium and high risk of bias, respectively (Table 1). The authors present the “risk of bias” in Table 1.

**Table 1:** Assessment of study bias.

Authors Domains	D1	D2	D3	D4	D5	D6	D7	D8	D9	D10	Total
Kurhaluk and Tkachenko^13^	0	2	0	1	1	2	2	2	2	2	14
Baltaci et al.^14^	0	2	0	1	1	2	1	1	0	1	9
Bojková et al.^15^	0	2	2	1	1	1	1	2	2	0	12
González et al.^16^	0	2	0	2	1	1	2	2	2	0	12
Moselhy et al.^17^	0	2	1	2	2	1	2	2	0	2	14
Sáez et al.^18^	0	2	0	1	1	1	2	2	0	0	9
Cos et al.^19^	1	2	0	1	1	1	2	2	2	0	12
el-Aziz^20^	0	2	0	0	0	1	2	2	0	2	9
Lenoir et al.^21^	1	1	0	1	1	1	1	2	0	0	8
Saez et al.^22^	0	1	0	1	1	1	1	2	0	0	7

Low risk = 2; Medium risk = 1; High risk = 0.

These studies were conducted in Poland, Slovakia, Turkey, Spain, Egypt, Germany, the United States, and the United Kingdom. The publications were published between 2005 and 2022. In total, 756 animals were studied. The number of tumor-bearing animals in each study varied between 48 and 126 (Table 2).

**Table 2:** Data collected from manuscripts selected for the systematic review.

Authors	Year	Country	Number of Animals	Animal model	Treatment isolated or associated	Treatment period (week)	Substance used in tumor induction	Exposition time	Mean dose	Apoptosis evaluated?	Reduction of the tumor
Kurhaluk and Tkachenko[13]	2022	Poland	154	Sprague-Dawley rats	With Metformin	16	NMU and DMBA	Light/dark cycle 12h/12h	1.8 mg/Kg/day	Yes, apoptotic marker not available	Yes
Baltaci et al.^14^	2017	Türkiye	42	Wistar rats	With Zinc	8	DMBA	Light/dark cycle 12h/12h	0.2 mg/Kg/day	none	No
Bojková et al.^15^	2017	Slovakia	82	Sprague-Dawley rats	With Metformin	14	DMBA	Light/dark cycle 12h/12h	1.6 mg/Kg/day	Cleaved caspase-3, BAX, BCL-2	Yes
González et al.^16^	2010	Spain	60	Sprague-Dawley rats	Alone	8	DMBA	Light/dark cycle 12h/12h	0.1 µg/kg/day	None	No
Moselhy et al.^17^	2008	Egypt	48	Sprague-Dawley rats	With Lycopene (Lyco)	30	DMBA	Light/dark cycle 12h/12h	2.5 mg /Kg/day	Yes, apoptotic marker not available	Yes
Sáez et al.^18^	2007	Spain	100	Sprague-Dawley rats	Alone	9	DMBA	1 ½ h before dark	20 mg/Kg/day	NK cells quantification	No
Cos et al.^19^	2006	Spain	60	Sprague-Dawley rats	With testosterone	9	DMBA	Light/dark cycle 12h/12h	1mg/Kg/day	None	Yes
el-Aziz et al.^20^	2005	Egypt	80	Sprague-Dawley rats	with retinoic acid (RA) and Nigella Sativa (NS)	12	DMBA	3 h before sunset	20 mg/kg/day	Caspase-3, percentage of DNA fragmentation and TNF-alpha	Yes
Lenoir et al.^21^	2005	France	60	Sprague-Dawley rats	Aone	24	DMBA	3 h before the lights were turned off	10 mg/kg/day	None	Yes
Saez et al.^22^	2005	Spain	70	Sprague-Dawley rats	Alone	16	DMBA	1/2 h before the onset of dark	20 mg/Kg/day	None	No

ML, Melatonin; NMU, N-methyl-N-nitrosourea; DMBA, 7,12-Dimethylbenz[a] Anthracene; NA, Not Applicable, data not presented in this study.

Melatonin alone was used in four of the ten studies (Table 2). In the others, melatonin was associated with zinc, lycopene, retinoic acid, “Nigella Sativa”, or metformin. In eight studies, melatonin was orally administered (gavage or through water in the drinking fountain), and in two studies, it was injected intraperitoneally.

In four of these 10 studies, melatonin treatment was performed between 1 h and 3 h before the start of the dark period. The treatment period and melatonin dosage used were, respectively, between 8 and 30-weeks, while the mean dose over 1.6 mg/kg/day was used in 7 of 10 studies. Other dosages were inferior to 1 µg/Kg/day (Table 2).

From the studies analyzed, six presented a reduction in tumors as a correlation with the apoptosis process, although four studies did not observe this effect. Likewise, studies performed by Baltaci et al.,^14^ González et al.,^16^ and Saez et al.^22^ did not demonstrate evidence of tumor reduction or apoptotic or antitumor action of melatonin (Table 2). Baltaci et al.^14^ and González et al.^16^ used low doses of melatonin (Table 2).

## Discussion

The oncostatic mechanisms of melatonin action may include anti-proliferative, anti-angiogenic, apoptosis-inducing, invasion, metastatic inhibition, and increased modulation of the immune system.^23^ The present review shows that melatonin inhibited tumorigenesis in three studies with the lowest risk of bias, which reinforces the oncostatic action of melatonin. However, several studies have combined other substances to evaluate their oncostatic action, making it difficult to infer the mechanisms involved.

Melatonin regulates the reproductive system, ovarian activity, and hormonal production, especially estrogen.^24^ Furthermore, it is related to inhibiting angiogenic factor expression in both *in vitro* and *in vivo* breast cancer studies.^25^

Studies have shown that melatonin may have an apoptotic effect by increasing the expression levels of Fas, Fas-L, and p-53 proteins, together with an increase in the expression of Bcl-2.^10^ These data suggest that melatonin can modulate the apoptotic process, since the expression of pro- and anti-apoptotic factors can be affected by this hormone.^9^^,^^10^ Notably, some of these proteins initiate apoptosis when the process can be reversed.^10^ However, the mechanisms involved in controlling the expression of these genes, as well as those involved in the final apoptosis cascade (cleavage by caspase-3, for example), require further investigation.

Other important anticancer effects of melatonin reported in the literature include the reduction of migration and invasion of tumor cells, and these properties are related to the action of melatonin on the cytoskeleton, as demonstrated in breast cancer cells.^25^ However, experimental studies in cancer models have shown that the effects of melatonin on tumor development are approximately 40 %, and that the main pathway for tumor reduction is cellular apoptosis. In addition, tumor cells are inhibited from transitioning from the G1 phase to the S phase of the cell cycle. These data can be explained by the quality of the studies, as 60 % were classified as having a high or moderate risk of bias. The SYRCLE tool identified this risk of bias as primarily due to inadequate randomization, blinding, or attrition reporting (Table 1). This, coupled with methodological heterogeneity ‒ such as divergent doses (0.1 µg–20 mg/kg/day), routes (oral/injected), and timing (day vs. circadian-synchronized administration) ‒ may explain inconsistent results. Notably, the three studies with the lowest bias unanimously reported tumor suppression, underscoring the importance of standardized protocols in further studies.

The physiological concentration of melatonin can vary in picomolar concentrations and is more pronounced at night and in the ovarian follicle during ovulation.^8^ In mammals, melatonin modulates physiological functions by activating at least two pharmacologically and molecularly distinct receptors, MT_1_ and MT_2_.^8^ In addition, excess melatonin can reduce the expression of melatonin receptors and lead to receptor-independent effects.^8^^,^^9^^,^^26^ As for its amphiphilicity, melatonin is able to cross the cell, organelles, and nuclear membranes and directly interact with intracellular molecules in the so-called non-receptor-mediated actions.^26^ Melatonin and its metabolites act as direct free radical scavengers, upregulating antioxidative enzyme transcription and activity, and binding to transition metals to prevent the production of hydroxyl radicals. Furthermore, because melatonin is highly concentrated in the mitochondria, it shields DNA, lipids, and proteins from oxidative damage. The mechanisms of the antioxidant action of melatonin have been extensively reviewed elsewhere.^26^ Therefore, this action explains part of the tumor reduction. Two studies did not confirm tumor reduction when a mean melatonin dose of 20 mg/kg/day was administered. In such cases, the quality of the study may have interfered with the results.^18^^,^^22^ A possible explanation for the results of these studies is that extremely high doses (20 mg/kg/day) are supraphysiological doses that may downregulate MT1/MT2 receptor expression,^13^^,^^26^ limiting receptor-mediated effects. Excessive concentrations can also trigger nonspecific effects, which may paradoxically affect oxidative stress. In addition, Sáez et al.^18^ showed that animals subjected to exercise-induced stress could activate proinflammatory pathways that counteract melatonin’s antiproliferative effects.

Generally, in clinical practice, higher doses of melatonin are required to observe an antiproliferative effect. However, in any case of chronic treatment, it should be kept in mind that melatonin should always be administered at night, and its plasma concentration should not increase early in the morning.^26^ In addition, supraphysiological doses (e.g., 20 mg/kg/day) may downregulate melatonin receptors or trigger nonspecific effects, as noted in studies by Sáez et al.^18^ and Kurhaluk et al.^13^ Variability in animal metabolism and circadian timing could further explain inconsistent results.

This review highlights the key limitations in the extant literature: (1) Methodological heterogeneity (doses, routes, and timing), (2) Bias risks (unblinded designs and attrition gaps), and (3) Reliance on DMBA models that may not recapitulate human diseases. Notably, only rigorously designed (low-bias) studies have consistently reported tumor suppression, underscoring the need for standardized protocols. Further studies should address metastasis, control circadian variables, and clarify the mechanisms of melatonin independent of adjunct therapies. In addition, there were some language barriers, and studies in Russian, Chinese, and Arabic were excluded. Given the methodological diversity (e.g., melatonin combined with other substances, differing tumor induction methods), a meta-analysis was deemed inappropriate. The moderate-to-high risk of bias in 60 % of included studies ‒ primarily due to inadequate randomization, blinding, or attrition reporting ‒ limits the reliability of pooled conclusions. Notably, only low-bias studies consistently demonstrated melatonin's tumor-suppressive effects, underscoring the influence of methodological rigor on outcomes. Future research should prioritize standardized designs with stringent bias controls to strengthen translational validity.

## Conclusion

The literature shows that in animal models of breast tumors, high doses of melatonin can induce tumor reduction by cell death through apoptosis. However, further studies are required to understand the mechanisms by which this hormone acts in mammary carcinogenesis. Key aspects that could be more thoroughly addressed in future research include prioritizing tumor models that better reflect the complexity of human breast cancer; establishing standardized melatonin dosing regimens and administration routes; controlling circadian timing in treatment protocols and others.

## Ethics approval and consent to participate

Not applicable.

## Consent for publication

Not applicable.

## Availability of data and materials

All data generated or analyzed during this study are included in this published article.

## Authors' contributions

1) Conceptualization: JMSJ, ECB, and JCN; 2) Methodology: JMSJ, ICES, and CFS; 3) Investigation and data curation: LHCMB and ISF; 4) Formal analysis: JMSJ, MSA, and LLD; 5) Validation: JMSJ, LLD, and ICES; 6) Writing-original draft preparation: MSA and LLD; 7) Writing-review and editing: JMSJ, MSA, and LLD; 8) Supervision: JMSJ, ECB, and JCN.

## Funding

“Fundação de Amparo à Pesquisa do Estado de São Paulo” (FAPESP 2018/24224-9).

## Data availability

The datasets generated and/or analyzed during the current study are available from the corresponding author upon reasonable request.

## Declaration of competing interest

The authors declare the following financial interests/personal relationships which may be considered as potential competing interests: Mayara Souza Alves reports was provided by Universidade de São Paulo. If there are other authors, they declare that they have no known competing financial interests or personal relationships that could have appeared to influence the work reported in this paper
